# Continuous venovenous haemofiltration with citrate-buffered replacement solution is safe and efficacious in patients with a bleeding tendency: a prospective observational study

**DOI:** 10.1186/1471-2369-14-89

**Published:** 2013-04-18

**Authors:** Shaikh A Nurmohamed, Borefore P Jallah, Marc G Vervloet, Gul Yldirim, Pieter M ter Wee, AB Johan Groeneveld

**Affiliations:** 1 Departments of Nephrology, VU University Medical Centre, Amsterdam, The Netherlands; 2 Departments of Intensive Care, VU University Medical Centre, Amsterdam, The Netherlands

**Keywords:** Continuous venovenous haemofiltration, Citrate, Replacement solution, Efficacy, Safety

## Abstract

**Background:**

There is ongoing controversy concerning optimum anticoagulation and buffering in continuous venovenous haemofiltration (CVVH). Regional anticoagulation with trisodium citrate also acting as a buffer in the replacement fluid has several advantages and disadvantages over prefilter citrate administration alone. We analysed a large cohort of patients with acute kidney injury (AKI) treated by the former method and hypothesized that it is safe and efficacious.

**Methods:**

Patients admitted at the intensive care unit with AKI and a high bleeding risk, without exclusion of liver disease, treated by CVVH with citrate in a custom-made replacement solution were prospectively included. Patient and CVVH characteristics, including citrate accumulation, were evaluated in outcome groups. A standardized mortality rate (SMR) was calculated using the simplified acute physiology score II.

**Results:**

Ninety-seven patients were included; metabolic control was adequate and did not differ between outcome groups, apart from lower pH/bicarbonate in non-survivors. Citrate accumulation was proven in 9% and was timely identified. These patients had about threefold higher plasma transaminases and higher CVVH dose and mortality. The hospital mortality was 60% with a SMR of 1.1 (95% confidence interval 0.90-1.40): age and hyperlactatemia, rather than CVVH-characteristics and citrate accumulation, predicted mortality in multivariable analysis.

**Conclusion:**

In critically ill, patients with AKI at high risk of bleeding, CVVH with citrate-containing replacement solution is safe and efficacious. The risk for citrate accumulation is 9% and best predicted by levels of transaminases. It carries, when citrate is discontinued, no attributable mortality.

## Background

Despite improvements in therapy, the mortality rate of critically ill patients with acute kidney injury (AKI) remains 50% or higher. Continuous renal replacement therapy (CRRT) carries the need for continuous anticoagulation to maintain circuit patency whereas clotting may contribute to ineffective treatment and blood loss. However, systemic anticoagulation may contribute to bleeding complications in 5-26% of patients [1]. Citrate has been widely used for conventional haemodialysis and has been successfully adapted for regional anticoagulation in (various modes of) CRRT [2-18]. Citrate offers an anticoagulant effect through its ability to chelate calcium; it acts regionally when administered prefilter and thus reduces the risk of bleeding as compared to systemic anticoagulation [1]. Citrate is cleared by the tricarboxylic acid pathway in the liver and other organs, thereby producing bicarbonate, so that it can also act as a buffer.

The most frequently used method employs hypertonic trisodium citrate [2,4-7,15] at the entry of the filter together with the use of hypotonic low sodium alkali-free replacement solution in postdilution mode for continuous venovenous haemo(dia)filtration (CVVH). Although reported not to increase and perhaps even to decrease mortality [15], the use of hypertonic citrate solutions [4,8,13,15,19-21] carries a potentially higher risk of metabolic derangements, including hypernatremia and citrate accumulation with hypocalcemia. However, it should be underlined that these complications are rarely observed in absence of protocol violation or accidental overdosing. The use of isotonic citrate solutions [7,18] prevents the risk of hypernatremia while the risk of citrate accumulation, although potentially lower, is generally not related to the concentration of the citrate solution but more strictly dependent on the citrate dose adopted. However, the risk factors for accumulation vary across these studies, whereas accumulation may be independently associated with mortality [17,20]. Accumulation of calcium-citrate complexes results in an increase of the total to ionised calcium ratio and, if the metabolism of citrate fails, a high anion gap acidosis. Metabolic alkalosis may also develop when too much citrate enters the circulation and is adequately metabolized, and may also contribute to a downhill course [2,22,23]. The second method, adopted by us from relatively small prior studies, employs citrate-containing replacement solution in predilution mode of CVVH, which is isotonic and acts as an anticoagulant and buffer, so that the amount of bicarbonate equivalents and control of acid–base status are similar to that employed when lactate- or bicarbonate-buffered solutions are used [3,8,10-12,24-26]. In two recent systematic reviews on regional citrate (vs heparin) anticoagulation, six randomized clinical trials, of which only two employed citrate in predilution replacement solution as we did, were pooled, but an effect on mortality remained unclear because of paucity of data [27,28]. Moreover, many of these studies included patients without a bleeding tendency and excluded those with liver disease [26], so that the effect of citrate-CRRT on mortality of patients with those risks remains obscure.

For this prospective observational study, we hypothesized that CVVH with citrate as anticoagulant and buffer in replacement solution is safe and efficacious in patients with a bleeding tendency, without excluding those with liver disease. We therefore evaluated metabolic control of the system, including citrate accumulation and risk factors thereof, and their impact on patient outcome.

## Methods

We introduced the treatment as a standard after approval by the institutional ethics committee (VU University medical ethical committee, reference number: 2003/187) and do not require, by Dutch law (Medical Research Involving Human Subjects Act (WMO)) informed consent for the anonymous collection of data obtained during standard monitoring of this treatment.

A modified version of the CVVH with citrate in replacement fluid originally described by Palsson and Niles was used [3], after introduction in our hospital in 2005 [25]. This solution contains trisodium citrate (13.3 mmol/L), sodium (140 mmol/L), chloride (104 mmol/L), potassium (3.0 mmol/L), glucose (5.0 mmol/L) and magnesium (0.5 mmol/L). All consecutive patients in a two year period admitted at the intensive care unit (ICU) of our university hospital with AKI treated by CVVH with citrate-containing replacement solution for clinical reasons were prospectively included in this observational study. Reasons included a bleeding tendency, arbitrarily defined as a platelet count below 40 × 10^9^/L, an activated partial thromboplastin time (aPTT) of more than 60 sec or a prothrombin time (PT, international normalised ratio INR) of more than 2.0, a recent major bleeding. There were no exclusion criteria. The CVVH was started and stopped at the discretion of the treating intensive care physician and consultant nephrologist.

### Treatment

Vascular access was secured by inserting an 11 F double lumen catheter (GamCath. Gambro, Germany) into the jugular, femoral or subclavian vein. CVVH was carried out using a haemofiltration machine (DIAPACT, B Braun, Melsungen, Germany). In all patients a 1.9 m^2^ highly permeable cellulose triacetate haemofilter was used (NIPRO UF205, Nissho corporation, Japan). Filters were routinely changed after 72 hours. All patients were treated by CVVH in the predilution mode. The blood flow was set at 180 ml/min. The citrate-containing replacement solution ran at a rate of 2400 ml/h, and the rate of infusion of the citrate-based solution was continuously coupled to the blood flow. The replacement solution was infused after the blood pump in order to prevent backflow to the patient. Patients had a separate intravenous infusion pump to administer calcium glubionate (Calcium Sandoz^®^ containing calcium 0.225 mmol/ml, Novartis, The Netherlands). The rate of calcium administration was adjusted to keep the ionized calcium concentration between 0.90 and 1.10 mmol/L. Calcium-levels in the extracorporeal circuit were not measured since they are almost universally below 0.4 mmol/L.

### Safety monitoring and criteria to stop CVVH with citrate

Total to ionized calcium concentration ratios, pH, bicarbonate, base excess and anion gap were measured or calculated at least four times daily in blood samples, drawn from an arterial catheter, to prevent complications such as citrate accumulation, low systemic ionized calcium, high anion gap acidosis or metabolic alkalosis. The first measurement was done one hour after initiation of CVVH. Blood gas analysis was performed every 6 h on the ICU, using a Bayer RapidLab 865 Blood Gas Analyser (Bayer, Leverkusen, Germany), or in the hospitals clinical laboratory, using a Radiometer ABL800 Flex (Radiometer, Copenhagen, Denmark). A multichannel analyzer was used to measure six hourly the levels of sodium (n 136–146 mmol/l), potassium (n 3.6-4.8 mmol/l) and chloride (n 98–108 mmol/l) (Hitachi Modular ISE 900, Roche Diagnostics, Mannheim, Germany) and the levels of total calcium (n 2.20-2.60 mmol/l), magnesium (n 0.70-1.00 mmol/l), albumin (n 35–52 g/l), phosphate (n 0.70-1.40 mmol/l), lactate (n <2.2 mmol/l), creatinine (n 60–110 μmol/l) and urea (n 3.0-7.5 mmol/l) (Hitachi Modular P800, Roche Diagnostics, Mannheim, Germany). Afterwards, these measurements were done 6-hourly. Lactate and alkaline phosphatase, gamma glutamyl transpeptidase, transaminase plasma levels (Method traceable to IFCC method, Modular analytics, Roche diagnostics, Mannheim, Germany) and bilirubin (DPD method, Modular analytics, Roche diagnostics, Mannheim, Germany) were measured at least once daily in the morning. The safety monitoring lasted for the duration of CVVH treatment. Citrate CVVH was stopped, if the patient fulfilled one of the following criteria for accumulation, in accordance with the literature [12,18-20]: total to ionized calcium ratio of more than 2.5, clinical signs of hypocalcemia (tetanic symptoms or prolonged QT interval), persistent metabolic alkalosis with a base excess of more than 10 mmol/l, or progressive non-lactic acidosis (pH < 7.20) combined with an increase in anion gap (>13 mmol/l), in the absence of another explanation than citrate. If there were signs of citrate accumulation, predilution CVVH was continued using bicarbonate-based replacement solution without anticoagulation (ionized calcium concentration of 1.75 mmol/l). A new episode of CVVH was defined as a period of at least 48 hours between the stop of CVVH treatment and the beginning of a new CVVH course.

### Data collection

We designed a predefined checklist for this prospective observational study focusing on safety and efficacy issues. Our ICU has an electronic patient file where patients’ details are stored. Baseline characteristics were retrieved, including age, gender, weight, height, reason of admission, medical history, coagulation, bleeding and renal function. A severity of illness score at the time of ICU admission was generated by the Acute Physiology and Chronic Health Evaluation (APACHE II) score. The sequential organ failure assessment (SOFA) score was obtained at admission, day 3 and at the day of start CVVH. Safety and efficacy were evaluated by metabolic control, potential complications and their impact on hospital mortality, by comparing survivors and non-survivors. A standardized mortality rate (SMR) to compare observed with expected hospital mortality was calculated using the simplified acute physiology score II (SAPS II). Data on safety included course of total and ionized calcium, the total to ionized calcium ratio and the anion gap, the frequency of change in the calcium pump and of citrate accumulation, bleeding complications and hospital mortality rates. Data concerning efficacy were retrieved such as filter life, reason of filter termination, azotaemic control determined by course of plasma creatinine and urea and acid–base control judged from course of pH and bicarbonate levels during treatment. Prescribed dose was defined as the total ultrafiltration volume prescribed per kilogram preadmission body weight per hour; it was averaged per day and did not take down time into account. As all patients were treated in the predilution mode, the ultrafiltration flow per hour (Quf) was adjusted by the following formula: [Qb × 60 × (1-Ht)] × Quf/[(Qb × 60 × (1-Ht)) + Qs], where Qb = blood flow per minute and Qs = substitution flow per hour.

### Statistical analysis

The data were mostly normally distributed (Kolmogorov-Smirnov test P > 0.05) and values are summarized as mean ± standard deviation. The independent sample t-test was used for continuous variables and the Fisher’s exact test for categorical data. Generalized estimating equations, taking repeated measurements in the same patients into account, were used to evaluate overall differences between groups in time. We performed multiple logistic regression using backward elimination to assess the independent value of patient and CVVH characteristics to predict hospital mortality, including variables reaching statistical significance in univariate analyses (P < 0.05), and citrate accumulation was forced into the model. The odds ratio and its 95% confidence interval (CI) were calculated. The Hosmer-Lemeshow test was done to assess goodness-of-fit. Exact P values are given, unless <0.001, and considered statistically significant if <0.05.

## Results

A total of 97 patients were treated by citrate-CVVH during the study period. Five patients had two episodes of CVVH. Baseline characteristics of all patients are shown in Table 1. The survivors had a longer duration of ICU admission as compared to non-survivors, 22 ± 16 days and 14 ± 14 days (P = 0.02) respectively. The hospital mortality of all patients treated by citrate CVVH was 60%. The calculated expected mortality using the SAPS II score was 55%. With an overall SMR of 1.1 (95% CI: 0.90-1.40), observed mortality in this high bleeding risk study group was comparable to what is expected. Multiple logistic regression revealed that advancing age and high initial lactate level were associated with hospital mortality, without contributions of citrate accumulation, azotaemic control, acid–base balance and other patient and CVVH characteristics (X^2^ 12.1, df8, P = 0.15).

**Table 1:** Baseline characteristics

	**Survivors**	**Non-survivors**	**P**
	**n = 39**	**n = 58**	
Age (years)	57 ± 16	66 ± 15	0.007
Male (%)	25 (64)	42 (72)	0.36
Weight (kg)	79 ± 14	76 ± 18	0.29
APACHE II	23 ± 5	24 ± 7	0.31
SAPS II	52 ± 9	57 ± 13	0.04
SOFA day 1	12 ± 3	12 ± 4	0.74
SOFA day 3	13 ± 4	14 ± 3	0.27
SOFA at start CVVH	13 ± 4	14 ± 3	0.27
Manifest bleeding (%)	18 (46)	24 (41)	0.65
Blood transfusion (%)	21 (54)	32 (55)	0.90
Reason of admission (%)			0.27
Postoperative	18 (46)	18 (31)	
Respiratory insufficiency	10 (26)	25 (43)	
Cardiogenic shock	9 (23)	10 (17)	
After CPR	1 (3)	3 (5)	
CVVH	1 (3)	2 (3)	
Reason of renal failure (%)			0.10
Sepsis	8 (21)	14 (24)	
Ischemic	19 (49)	35 (60)	
Toxic	1 (3)	0	
Metabolic	2 (5)	0	
Auto-immune	2 (5)	1 (2)	
Unknown	3 (8)	3 (5)	
History of CKD	4 (10)	5 (9)	
At start of CVVH			
Creatinine (μmol/l)	364 ± 186	312 ± 233	0.28
Urea (mmol/l)	20.0 ± 7.2	21.1 ± 12.0	0.63
Potassium (mmol/l)	4.8 ± 0.8	4.5 ± 0.8	0.10
pH	7.33 ± 0.11	7.30 ± 0.10	0.12
Bicarbonate (mmol/l)	19.6 ± 3.9	18.6 ± 4.2	0.24
Lactate (mmol/l)	2.9 ± 2.2	4.3 ± 4.0	0.09
Diuresis (ml/day)	547 ± 709	601 ± 622	0.75
aPTT (sec)	50 ± 34	55 ± 30	0.51
PT-INR	1.63 ± 0.56	1.88 ± 0.82	0.14
Platelets (×10^9^/l)	116 ± 75	107 ± 72	0.89

Data are expressed as mean (±standard deviation) or number (percentage) of patients where appropriate. APACHE II Acute Physiology and Chronic Health Evaluation; SOFA, Sequential Organ Failure Assessment; SAPS II: Simplified Acute Physiological Score; CVVH: continuous venovenous haemofiltration; ICU: intensive care unit; CPR: cardiopulmonary resuscitation; CKD: chronic kidney disease; aPTT: activated partial thromboplastin time; PT-INR: prothrombin time–international normalized ratio.

### Metabolic control

**Figure 1:**
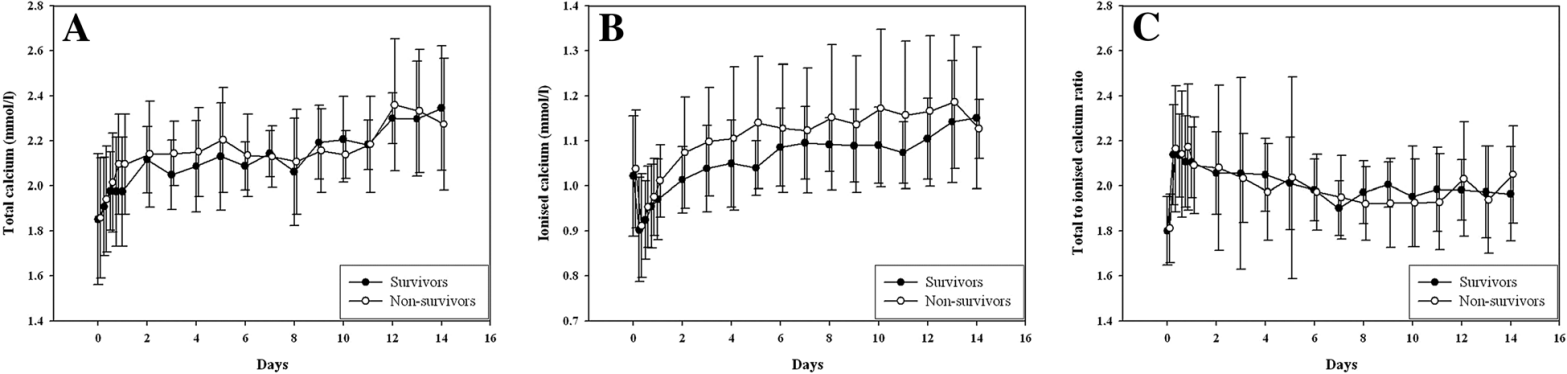
**Course of total calcium (A), ionised calcium (B) and total to ionised calcium ratio (C) during treatment by continuous venovenous haemofiltration with citrate.** The course of these calcium parameters was similar in survivors and non-survivors (P = 0.45 for total calcium, P = 0.38 for ionised calcium and P = 0.83 for total to ionised calcium ratio). Mean ± SD.

After initiating CVVH with citrate, ionized calcium decreased within a few hours and total calcium concentration gradually increased, irrespective of outcome (Figure 1). After adjustment of the calcium pump the ionized calcium concentration slowly increased. The total to ionized calcium ratio initially increased but stabilized in the first day. As demonstrated in Table 2, relatively more adjustments of the calcium pump were made in the first 48 hours of treatment as compared to the period thereafter; there were no differences between outcome groups. The mean calcium administration per hour of CVVH was 97 ± 65 mg (2.4 ± 1.6 mmol).

**Table 2:** Characteristics of continuous venovenous haemofiltration with citrate

	**Survivors**	**Non-survivors**	**P**
	**n = 39**	**n = 58**	
Blood flow (ml/min)	180 ± 0	180 ± 0	1.00
Substitution flow (ml/h)	2403 ± 16	2397 ± 59	0.54
Prescribed dose (ml/kg//h)	24 ± 4	26 ± 6	0.13
Filter life (h)	38 ± 24	23 ± 20	0.004
Reasons for termination (%)			0.05
Clotting	41	47	
Transfer to OR/radiology	13	4	
Renal function recovery	24	9	
Death of patient	0	14	
Elective change	15	10	
Citrate toxicity	1	7	
Miscellaneous	6	13	
Citrate accumulation	1	8	0.04
Changes in calcium infusion pump rate,			
day 1	2.2 ± 1.3	2.2 ± 1.5	0.99
day 2	1.6 ± 1.6	2.0 ± 2.0	0.37
day 3	1.5 ± 1.4	1.6 ± 1.2	0.70
day 5	1.6 ± 1.3	1.2 ± 1.1	0.99
day 7	1.9 ± 1.5	1.1 ± 1.1	0.18
Duration of CVVH (h)	219 ± 237	223 ± 507	0.96

Data are expressed as mean (± standard deviation) or number of patients where appropriate; CVVH: continuous venovenous haemofiltration.

**Figure 2:**
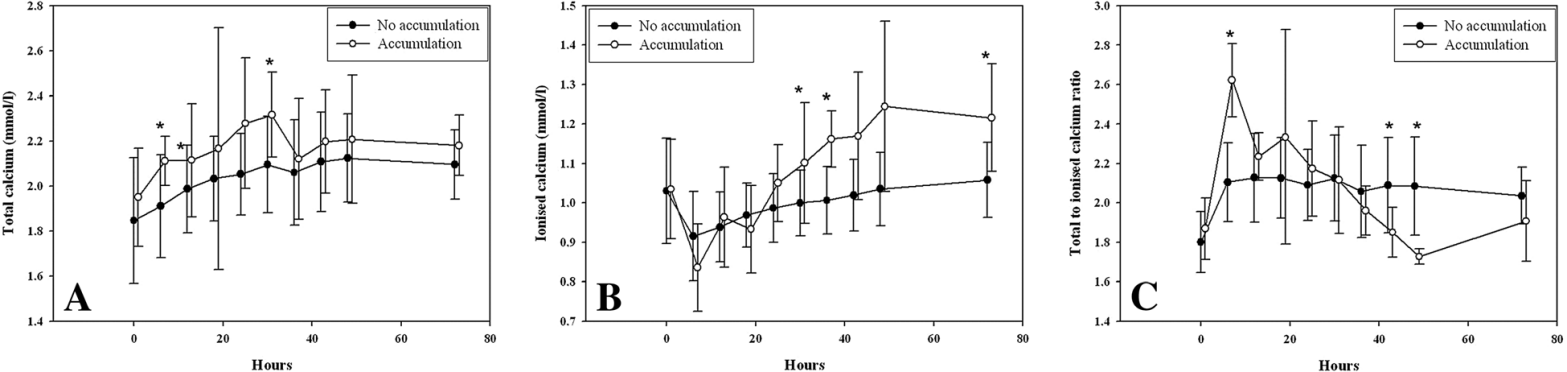
**Course of total calcium (A), ionised calcium (B) and total to ionised calcium ratio (C) during treatment by continuous venovenous haemofiltration in patients with and without citrate accumulation.** Patients with accumulation were converted to continuous venovenous haemofiltration with bicarbonate-based replacement solution without anticoagulation after a mean time of 13.0 ± 8.7 h. The differences between the groups reached significance at several time points (*P =0.05 or lower) with higher ionized calcium concentrations in patients with citrate accumulation after switching from citrate to bicarbonate-based replacement fluid (with ionized calcium concentration of 1.75 mmol/l), while the overall course of these calcium parameters was similar (P = 0.21 for total calcium, P = 0.92 for ionised calcium and P = 0.08 for total to ionised calcium ratio). Mean ± SD.

**Figure 3:**
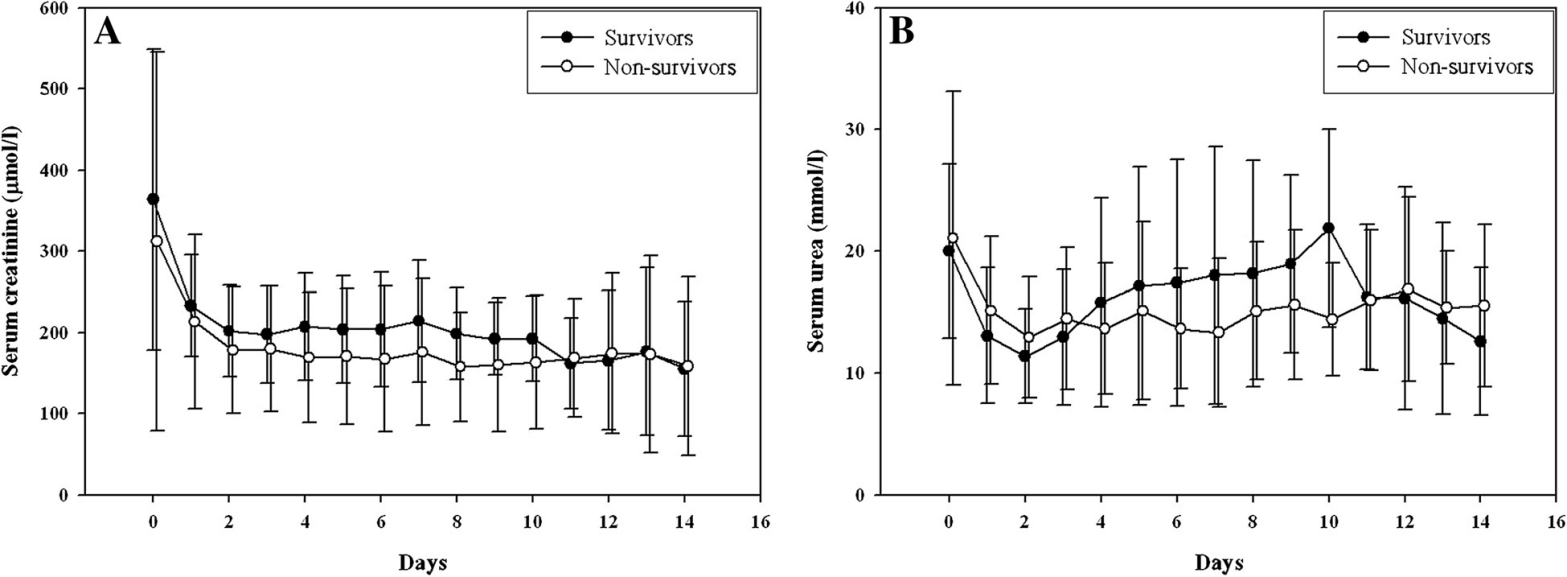
**Course of serum creatinine (A) and urea (B).** The azotaemic control was similar in survivors and non-survivors (P = 0.22 for creatinine and P = 0.97 for urea). Mean ± SD.

**Figure 4:**
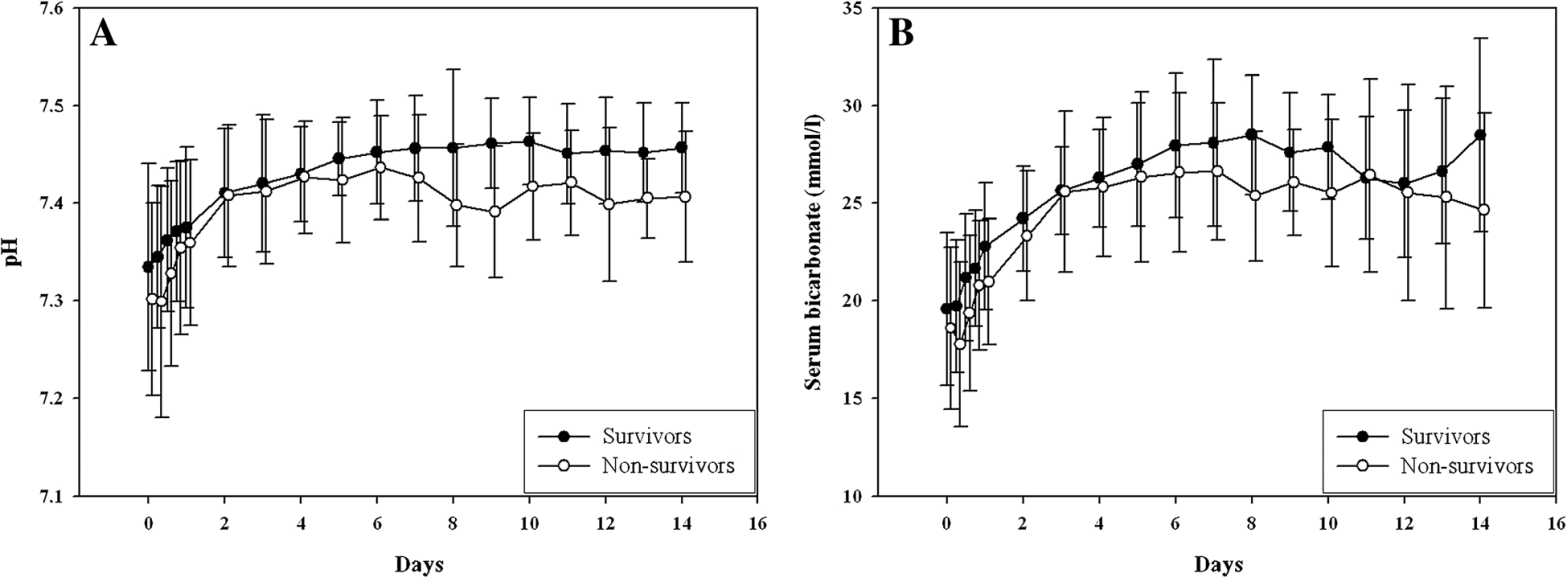
**Course of pH (A) and serum bicarbonate (B).** Both parameters are higher in survivors (P = 0.001 for pH and P = 0.003 for bicarbonate). Mean ± SD.

In 11 (11%) patients treatment with citrate was withdrawn because of suspected citrate accumulation; in two patients citrate was withdrawn because of an increasing total to ionized calcium ratio even though remaining below 2.5. Nine patients thus had proven accumulation (Table 3), primarily in patients with chronic kidney disease or a presumed ischemic origin of AKI, rather than sepsis. Accumulation usually occurred within a day after start of treatment (mean time to accumulation 13.0 ± 8.7 h); after withdrawal of citrate the calcium parameters rapidly normalized (Figure 2). Patients accumulating citrate were characterized by a greater disease severity at start of CVVH, lower body weight, higher transaminase concentrations in plasma, higher prescribed CVVH dose and higher mortality. Until withdrawal of citrate, patients with citrate accumulation did not show a major increase in anion gap, although the serum bicarbonate level decreased from 19.3 ± 4 to 16.3 ± 4 mmol/l concomitantly with a decrease in the sodium level from 146 ± 7 to 142 ± 5 mmol/l in the first six hours. In ROC analysis, the optimum cutoff value for predicting accumulation was a SOFA score at initiation of CVVH of 14, a prescribed dose of 26 ml/kg/h, an alanine aminotransaminase of 1455 U/l and an aspartate aminotransaminase of 489 U/l. Citrate-CVVH resulted in adequate azotaemic control (Figure 3), again irrespective of outcome. Hypernatremia or severe metabolic alkalosis attributable to CVVH treatment was not observed. Serum bicarbonate and pH levels gradually normalized after start of CVVH (Figure 4); these parameters, however, were lower in non-survivors than in survivors. At initiation of CVVH, the lactate level was higher in non-survivors (Table 2); during the course of treatment, the lactate levels remained higher at several time points and predicted mortality (P < 0.001). Additional bicarbonate infusion was never required.

**Table 3:** Clinical and biochemical characteristics of patients with and without citrate accumulation

	**Citrate accumulation n = 9**	**No citrate accumulation n = 88**	**P**
Age (years)	69 ± 14	62 ± 16	0.18
Male (%)	5 (56)	62 (71)	0.36
Weight (kg)	62 ± 8	79 ± 16	0.004
APACHE II	23 ± 4	24 ± 7	0.49
SAPS II	56 ± 8	55 ± 12	0.79
SOFA day 1	13 ± 3	12 ± 4	0.11
SOFA day 3	16 ± 3	13 ± 4	0.08
SOFA at start CVVH	16 ± 2	13 ± 4	0.01
Duration of ICU admission (days)	12 ± 9	18 ± 15	0.08
Reason of admission (%)			0.07
Postoperative	1 (11)	35 (40)	
Respiratory insufficiency	5 (56)	30 (34)	
Cardiogenic shock	3 (33)	16 (18)	
After CPR	0	4 (5)	
CVVH	0	3 (3)	
Reason of renal failure (%)			0.61
Sepsis	0	22 (25)	
Ischemic	8 (89)	46 (52)	
Toxic	0	1 (1)	
Metabolic	0	2 (2)	
Auto-immune	0	3 (3)	
Unknown	0	6 (7)	
History of CKD	1 (11)	8 (9)	
Filter life (h)	12.4 ± 8.3	30.9 ± 23.1	0.0004
Prescribed dose (ml/kg/h)	30 ± 4	24 ± 5	0.001
At start CVVH			
pH	7.31 ± 0.08	7.32 ± 0.10	0.77
Lactate (mmol/l)	6.2 ± 3.6	3.5 ± 3.4	0.17
Anion gap (mmol/l)	22 ± 6	19 ± 6	0.13
Alanine aminotransaminase (U/l)	652 ± 1031	227 ± 424	0.03
Aspartate aminotransaminase (U/l)	1861 ± 3305	409 ± 942	0.01
Gamma-glutamyl transferase (U/l)	86 ± 97	86 ± 113	0.99
Alkaline phosphatase (U/l)	123 ± 74	132 ± 154	0.80
Bilirubin (μmol/l)	133 ± 133	53 ± 106	0.21
Lactate dehydrogenase (U/l)	1396 ± 1088	1626 ± 3336	0.73
PT-INR	1.75 ± 0.38	1.78 ± 0.77	0.84
Albumin (g/l)	19 ± 4	17 ± 5	0.32
Acid–base balance during CVVH pH			
t = 0 h	7.31 ± 0.08	7.32 ± 0.10	0.77
t = 6 h	7.30 ± 0.10	7.32 ± 0.11	0.60
t = 12 h	7.31 ± 0.08	7.35 ± 0.09	0.23
Anion gap (mmol/l)			
t = 0 h	22 ± 6	19 ± 6	0.34
t = 6 h	22 ± 5	21 ± 5	0.35
t = 12 h	20 ± 7	20 ± 6	0.96
Hospital mortality	6 (86)	50 (59)	0.02

Data are expressed as mean (±standard deviation) or number (percentage) of patients where appropriate. APACHE II: Acute Physiology and Chronic Health Evaluation; SOFA, Sequential Organ Failure Assessment; SAPS II: Simplified Acute Physiological Score; CVVH: continuous venovenous haemofiltration; ICU: intensive care unit; CPR: cardiopulmonary resuscitation; CKD: chronic kidney disease; PT-INR: prothrombin time–international normalized ratio.

## Discussion

Our data suggest that the treatment of critically ill AKI patients at high risk of bleeding by CVVH with citrate-containing replacement solution is safe and efficacious, since vital outcome was not related to citrate accumulation, azotaemic and acid-base control. Otherwise, this is the largest series on citrate in replacement fluid we are aware of (3,8,10-12). The technique we applied was introduced by Palsson and Niles [3], and the solution became commercially available in our country after completion of our studies [25,26]. In our earlier report we already demonstrated the superiority of this technique (n = 20) as compared to predilution anticoagulant free CVVH (n = 31) regarding filter life [25].

In this large cohort we primarily focused on safety and efficacy of citrate-containing replacement fluid. There was no attributable mortality as the SMR was approximately one, even in the high bleeding risk patients we included, thereby supporting the safety of the system. Otherwise, the hospital mortality of our patients is hard to compare with that of about 42-90% in other studies on citrate-CVVH, also including patients with less and more severe underlying disease or on CVVH in postdilution mode [3,10-13,15,16,18,20,24,27],[28]. The latter studies did not evaluate the contribution of citrate-CVVH-related parameters and metabolic control to mortality as we did. In one study, treatment with hypertonic citrate and CVVH in postdilution mode reduced mortality when compared to nadroparin as systemic anticoagulant [15]. In a recent trial, however, CVVH with citrate-containing replacement solution carried similar mortality when compared to CVVH with heparin [16]. The results of our Dutch multicenter randomized trial comparing citrate-containing replacement fluid with bicarbonate-containing fluid and heparin are awaited (NCT 0209378). There is no trial we are aware of comparing citrate-CVVH modes to fully appreciate the relative merits and detriments.

Some prior investigators have adopted the technique of regional anticoagulation with citrate added to the replacement solution in relatively small series and safety and efficacy appeared promising [3,10-12,24]. The latter were more elaborately confirmed in the current study. Application of this system may circumvent the hazards associated with potentially uncontrolled infusion of hypertonic citrate causing severe hypocalcemia with adverse cardiovascular events that may be fatal [2,19-23]. Hypernatremia, severe metabolic alkalosis or high anion gap acidosis which may also occur when trisodium citrate is accidentally overdosed with use of hypertonic solutions in the latter studies, was not seen in ours, using a isotonic replacement solution with less risk for overdosing, even though the citrate load per hour is only slightly less than resulting from a 70 ml/h infusion of a 0.5 molar citrate solution prefilter to reach similar anticoagulation. After initiating CVVH there was a gradual and adequate recovery of metabolic acidosis. The lower serum pH and bicarbonate in the non-survivors may be explained by the higher lactate levels, rather than by inadequate metabolic control by citrate-CVVH and acid-base balance did not independently predict mortality. The equivalence of citrate- with bicarbonate-buffer in CVVH replacement solution in this respect has been demonstrated by us before in a smaller study [26] and the current one underscores the potential of citrate in the replacement solution to act as an adequate and single buffer. The azotaemic control was adequate, even with replacement fluid in predilution mode as noted before [29], irrespective of patient outcome. In contrast, the occurrence of citrate accumulation with elevated total to ionized calcium ratio in about 10% of our patients was associated with increased mortality, in the absence of a significant increasing anion gap acidosis. However, patients accumulating citrate had greater disease severity at initiation of CVVH, whereas citrate accumulation did not independently contribute to mortality, in contrast to recent observations on a hypertonic system [12,20]. This can be explained in part by our safety monitoring protocol, allowing timely identification of accumulation and withdrawal of citrate, so that the total to ionized calcium ratio rapidly normalized. Otherwise, the calcium levels were kept within acceptable limits by only few calcium pump adjustments per day. The calculation of total to ionized calcium ratio proved to be a better parameter for citrate accumulation than anion gap.

In our study, severe liver abnormalities (especially more than ten to forty fold elevated transaminases) and, particularly, a relatively high prescribed CVVH dose (with more citrate loading) were major risk factors for citrate accumulation, as reported before in some [14,20] but not in other studies [17]. Durão et al. [13] suggested a prolonged PT as an indicator of liver dysfunction predicting citrate accumulation, but we could not confirm this. Kramer et al. [8] suggested feasibility of citrate anticoagulation in patients with advanced liver cirrhosis whereas predictors of accumulation were hard to identify. Our study suggests that ischemic AKI and perhaps ischemic hepatitis, but not minor liver abnormalities, may constitute a contraindication for citrate-CVVH unless dose reductions are applied to prevent citrate accumulation. In this setting, however, CVVH dose is coupled to citrate dose and harmful CVVH underdosing should be avoided when a citrate dose reduction is aimed at. The advantage, however, is that a separate citrate pump prefilter is not necessary with this system. We only provided the prescribed dose; in clinical practice the actually delivered dose is approximately 15% lower because of down times. If a rise in total to ionised calcium ratio to 2.5 does not level off within a few hours after reducing the dose of citrate-CVVH, bicarbonate-buffered replacement fluid is used without anticoagulation, in patients with a bleeding tendency admitted in our unit. This may also explain the reduced filter life in patients with citrate accumulation. The overall filter life of more than 30 h was adequate and comparable with that observed by others [3,11,16]. However, the filter life in non-survivors was approximately 15 hours shorter than in survivors, also reflecting the greater disease severity and associated clotting tendency in the former, which has been observed before [1].

Obviously, the limitations of our study include its observational nature with all inherent drawbacks such as the lack of randomisation. Our results should therefore be interpreted with caution. However, our observations in a relatively large group of high bleeding risk patients with a bleeding tendency, without excluding liver disease, extend the limited available data on safety and efficacy of this form of CVVH treatment [3,10-12,24-26].

## Conclusions

In conclusion, our results suggest that CVVH with citrate-containing replacement solution is safe and efficacious in patients with a bleeding tendency, even in the presence of moderately severe liver disease, provided that safety monitoring is strictly applied. The risk for citrate accumulation is about 9%. Marked elevations in transaminases and a high citrate-CVVH dose are major risk factors for citrate accumulation in mainly ischemia-associated AKI. Citrate accumulation may not contribute to mortality if timely recognized and followed by discontinuation of citrate.

## Competing interests

The authors declare that they have no competing interests.

## Authors’ contributions

BP Jallah and G Yldirim retrieved the data necessary for analysis, performed statistical analysis and drafted the initial manuscript. SA Nurmohamed initiated the study, participated in study design and data collection, and revised the manuscript. MG Vervloet and PM ter Wee were involved in drafting and revision of the manuscript. ABJ Groeneveld coordinated the study, participated in design of the study and statistical analysis, and was involved in writing the manuscript. All authors read and approved the final manuscript.

## Pre-publication history

The pre-publication history for this paper can be accessed here:

http://www.biomedcentral.com/1471-2369/14/89/prepub
